# The role of circulatory systemic environment in predicting interferon-alpha–induced depression: The neurogenic process as a potential mechanism

**DOI:** 10.1016/j.bbi.2019.06.018

**Published:** 2019-10

**Authors:** Alessandra Borsini, Carmine M. Pariante, Patricia A. Zunszain, Nilay Hepgul, Alice Russell, Zuzanna Zajkowska, Valeria Mondelli, Sandrine Thuret

**Affiliations:** a Department of Psychological Medicine, Institute of Psychiatry, Psychology and Neuroscience, King’s College London, UK; b Department of Basic and Clinical Neuroscience, Institute of Psychiatry, Psychology and Neuroscience, King’s College London, UK

**Keywords:** Serum, Hippocampal progenitor cells, Neurogenesis, Apoptosis, Depression, Inflammation, Interferon-alpha

## Abstract

Interferon (IFN)-α treatment for hepatitis C virus (HCV) is a well-recognized clinical model for inflammation-induced depression, but the brain cellular mechanisms underlying these effects are still not clear. Previous data reported an alteration in peripheral levels of inflammatory and neuroplasticity markers in the blood of depressed versus non-depressed patients. We investigated the *in vitro* effect of serum from depressed and non-depressed HCV patients (at baseline, before IFN-α; and after four weeks of IFN-α), on the apoptotic and neurogenic processes in a human hippocampal progenitor cells model. Results show that higher apoptosis during proliferation observed upon treatment of cells with baseline serum, and lower neuronal differentiation observed upon treatment with serum after 4 weeks of IFN-α, were predictive of later development of IFN-α–induced depression (odds ratio = 1.26, p = 0.06, and = 0.80, p = 0.01, respectively). While serum after IFN-α increased neurogenesis compared with baseline serum, a lower increase in neurogenesis was also predictive of later development of depression (odds ratio = 0.86; p = 0.006). Our results provide evidence for the fundamental role of the systemic milieu (captured by serum samples) in the regulation of hippocampal neurogenesis by inflammation, a putative mechanism involved in the development of neuropsychiatric conditions.

## Highlights

• The mechanisms underlying the effect of interferon (IFN)-alpha treatment for hepatitis C virus (HCV) are still not clear.
• We investigated the in vitro effect of serum from depressed and non-depressed HCV patients on human hippocampal neurogenesis.
• Lower neurogenesis upon treatment with serum were predictive of later development of depression.
• The systemic milieu exerts a fundamental role in the regulation of the neurogenic process.

## 1 Introduction

Approximately 200,000 individuals have chronic hepatitis C virus (HCV) in the UK, and 170 million worldwide (Harris et al., 2012). The current standard treatment for HCV infection involves subcutaneous injection with pegylated-interferon-alpha (peg-IFN-α), which clear the virus in up to 80% of patients while inducing a number of side effects (Wichers and Maes, 2002). Indeed, despite the efficacy of this treatment, IFN-α promotes depression in up to 35% of patients within the first 8 to 12 weeks of the therapy (Asnis and De La Garza, 2006, Capuron and Miller, 2004, Machado et al., 2017), as well as other side effects, including symptoms of anxiety, mania and fatigue (Sockalingam and Abbey, 2009). The brain cellular mechanisms underlying this association have not been completely elucidated yet.

Our work as well as previous evidence have reported that peripheral IFN-α acutely induces the production and release of several innate immune cytokine proteins (Hepgul et al., 2016, Raison et al., 2010). These cytokines are putatively involved in the depressogenic action of IFN-α. Higher serum or plasma levels of pro-inflammatory markers have been shown to be associated with an increased risk of major depressive disorder (Dowlati et al., 2010). Another study from our group provided evidence for increased inflammation in the blood of depressed patients when compared with healthy controls, together with an association between higher cytokine levels and lack of antidepressant response (Cattaneo et al., 2010). However, it is still unclear how these factors contained in peripheral blood can affect the brain and ultimately predispose the individual to the development of depression.

Several pathways through which peripheral blood factors, including inflammatory molecules, can be transmitted from the periphery to the brain have been investigated (Louveau et al., 2015). Previous studies have demonstrated that distinct cytokines, including interleukin-1beta (IL-1β), IL-6, and tumor necrosis factor-alpha (TNF-α), known to be upregulated both in the periphery and in the brain of patients with major depressive disorder (MDD), are able to increase blood brain barrier (BBB) permeability (Deli et al., 1995, Didier et al., 2003, Schwaninger et al., 1999) and ultimately to cause changes in discrete brain regions, including the hippocampus (Wang et al., 2008). This region is highly involved in the process of neurogenesis, which in humans principally occurs in the subventricular zone (SVZ) of the lateral ventricles and the sub-granular zone (SGZ) of the dentate gyrus (DG) of the hippocampus (Kempermann et al., 2018). Of relevance, previous research has demonstrated that peripheral administration of IFN-α or other cytokines, such as C-C motif chemokine ligand 11 (CCL11), induce depressive-like behaviours in rodents, affecting both cell proliferation and neuronal differentiation (Villeda et al., 2011, Villeda et al., 2014, Zheng et al., 2014). Taken together, these findings indicate that distinct factors contained in the circulatory systemic environment may affect the brain, and that the psychiatric side effects caused by IFN-α therapy might be a consequence of those factors to access the brain, and to disrupt neurogenesis or neuronal function in the hippocampus.

To investigate the contributing role of the systemic milieu in the regulation of hippocampal neurogenesis we used our previously established human hippocampal progenitor cell (HPC) line (Anacker et al., 2013a, Borsini et al., 2017b, Borsini et al., 2018, Zunszain et al., 2012), and examined the effect of treatment with serum samples from patients with HCV, before and after 4 weeks of IFN-α, on the neurogenic process. In particular, we investigated whether changes in proliferation, neurogenesis and apoptosis, caused by treatment with serum samples, were predictive of later development of IFN-α–induced depression.

## 2 Method

### 2.1 Patients cohort

The sub-group of patients, from which serum samples derived, is part of bigger cohort of patients recruited from the outpatient liver department of King’s College Hospital and St George’s Hospital. Eligible patients were adult patients with chronic HCV infection who were due to commence combination antiviral therapy with IFN-α and ribavirin without any additional direct-acting antiviral (boceprevir, telaprevir or simeprevir). The whole cohort of patients received combination therapy for at least 24 weeks. Patients were assessed at baseline when starting IFN-α treatment (TW0) and monthly for the first three months, at TW4, 8, 12, and 16 then two-monthly thereafter until the end of their treatment, at TW24. Combination therapy comprised of weekly subcutaneous IFN-α injections (1.5 µg per kg of body weight) and daily ribavirin tablets (800–1400 mg orally per day in 2 divided doses). Exclusion criteria for the larger cohort included, and for whom we had blood samples at both TW0 and TW4: a total of 33 eligible patients were included. The study was approved by the King’s College Hospital Research Ethics Committee (Ref: 10/H0808/30).

### 2.2 Demographics and clinical data of the IFN-α-treated HCV cohort

The Medical Research Council (MRC) Socio-demographic Schedule (Mallett et al., 2002) was administered at TW0 to collect socio-demographic data, including age and gender. The Mini-International Neuropsychiatric Interview (MINI) questionnaire (Sheehan et al., 1998) was administered at TW0, in order assess patients for a current depressive episode, as well as at follow-up assessments (TW4, 8, 12 and 24) to detect new onset cases of depression during IFN-α therapy. Demographic data shows that the selected cohort of 33 patients is predominantly characterised by male subjects (78.8%) with no significant difference in terms of age (Table 1). With respect to clinical data, 9 patients (27.3%) developed depression during IFN-α treatment, and 24 patients (72.7%) did not. Most importantly, the 9 patients developed depression between TW8 and TW24 (Table 2), that is, were not yet depressed at the time of the second (TW4) serum sample used for the present study.

**Table 1:** Characteristics of interferon-alpha (IFN-α) treated Hepatitis C (HCV) depressed vs. non-depressed patients assessed at baseline.

	Depressed *(n = 9)*	Non-depressed *(n = 24) (n* = 18)	p value
**Age (years)**			
Mean ± SEM	41.3 ± 5.3	41.6 ± 2.5	*p* = 0.9*
**Gender**			
Male	8 (88.8%)	18 (75%)	*p =* 0.4**

* Student’s *t*-test.

** Chi squared test.

**Table 2:** Development of depression during interferon-alpha (IFN-α) treatment.

		IFN-α–induced Depression		Total Number of Patients
		No	Yes	
DEPRESSION WEEK	None	24	0	24
	TW 8	0	5	5
	TW 12	0	2	2
	TW 16	0	1	1
	TW 24	0	1	1
Total Number of Patients		24	9	33

Treatment week (TW).

### 2.3 Human hippocampal progenitor cells

For the *in vitro* experiments with serum from IFN-α-treated HCV patients, multipotent human hippocampal progenitor cell line HPC0A07/03C, kindly provided by ReNeuron Ltd (Surrey, UK) was used. Cells were grown as previously described in RMM consisting of Dulbecco’s Modified Eagle’s Media/F12 (DMEM:F12, Invitrogen, Paisley, UK) supplemented with 0.03% human albumin (Baxter Healthcare, Compton, UK), 100 µg/mL human apo-transferrin, 16.2 µg/mL human putrescine diHCl, 5 µg/mL human recombinant insulin, 60 ng/mL progesterone, 2 mM L-glutamine and 40 ng/mL sodium selenite (Anacker et al., 2013a, Anacker et al., 2013b, Anacker et al., 2011, Borsini et al., 2017, Borsini et al., 2018, Zunszain et al., 2012). To maintain proliferation, 10 ng/mL human basic fibroblast growth factor (bFGF), 20 ng/mL human epidermal growth factor (EGF) and 100 nM 4-hydroxytamoxifen (4-OHT) were added. Cells were grown in 75 cm^2^ filtered cap culture flasks (Nunclon, Roskilde, Denmark) at 37 °C in 5% CO2 and regularly passaged at 80% confluence, until being transferred to plates for the differentiation experiments.

### 2.4 Proliferation, neurogenesis and apoptosis

In order to assess proliferation and apoptosis (during the proliferation stage) progenitor cells were plated on 96-well plates (Nunclon) at a density of 1.2 × 10^4^ cells per well per well in 100 μl RMM media. Cells were cultured in the presence of EGF, bFGF and 4-OHT for 24 h followed by 2 days incubation with 1% serum samples (the same concentration previously used in the lab as able to induce a profound effect without causing over-confluence or any alteration in the composition of neurons) from depressed or non-depressed IFN-α-treated HCV patients, also in the presence of growth factors containing 0.5 mg/ml penicillin streptomycin. The synthetic nucleotide bromodeoxyuridine (BrdU) (B5002, Sigma) with a final concentration of 10 μM was added to the media 4hrs before the end of the incubation to label proliferating cells.

Whereas, in order to assess neuronal differentiation and apoptosis (during the differentiation stage) cells were plated on 96-well plates (Nunclon) at a density of 1.2 × 10^4^ cells per well per well in 100 μl RMM media. Following the proliferation phase previously described, cells were washed twice for 15 min in RMM media (without EGF, bFGF and 4-OHT), and then cultured with 1% serum samples from depressed or non-depressed IFN-α–treated HCV patients in RMM containing 0.5 mg/ml penicillin streptomycin for subsequent 7 days.

### 2.5 Immunocytochemistry

At the end of the total incubation time, 2 days (proliferation) and 9 days (2 days proliferation followed by 7 days differentiation), cells were fixed with 4% paraformaldehyde (PFA) for 20 min at room temperature. Cells were then washed three times in phosphate-buffered saline (PBS) and stored at 4 °C in PBS in preparation for immunocytochemistry. Subsequently, PFA-fixed proliferating cells pulsed with BrdU were first incubated with 2 N hydrochloric acid for 25 min at room temperature followed by neutralisation with 0.1 M borate buffer for 10 min and 2 washes with PBS. Subsequently, both proliferating and differentiating cells were incubated in 50 µl of blocking solution constituted of 5% normal donkey serum (D9663, Sigma) in PBS containing 0.3% Triton X-100 (T8787, Sigma) for 1 h at room temperature. Primary antibodies for BrdU and Ki67 were used to assess proliferation (rat anti-BrdU, 1:500, Serotec; rabbit anti-Ki67, 1:500, Abcam). Antibodies for doublecortin (DCX) and microtubulin-associated protein-2 (MAP2) were used to assess neuroblasts and neurons respectively (rabbit anti-DCX, 1:500; mouse anti-MAP2, 1:500, Abcam), whereas antibody for caspase 3 (CC3) (rabbit anti-CC3, 1:500, Cell Signalling) were used to assess apoptosis and were diluted in blocking solution buffer. Finally, 30 µl/well were added and left at 4 °C overnight. The next day, cells were washed, incubated in blocking solution for 30 min and then with fluorescently tagged secondary antibodies for BrdU (Alexa Fluor 488 donkey anti-rat, 1:1000), Ki67 and CC3 (proliferation) (Alexa Fluor 555 donkey anti-rabbit, 1:1000), DCX and CC3 (differentiation) (Alexa Fluor 488 donkey anti-rabbit, 1:1000), and MAP2 (Alexa Fluor 555 donkey anti-mouse, 1:1000; all from Life Technologies) at 30 µl/well for 2 h at room temperature. After 3 washes with PBS, cells were stained with 0.02 mg/ml DAPI at 50 µl/well for 5 min and washed 3 more times.

### 2.6 Automated quantification of immunofluorescence

An unbiased and automated approach was employed to quantify cell number, cell proliferation, differentiation and apoptosis using the CellInsight* NXT High Content Screening (HCS) Platform (ThermoScientific). The specific paradigms for cellular markers were based on two BioApplications available in the Cell Insight machine software: (1) Target Activation, which allows for the quantification of nuclear markers (DAPI, BrdU and Ki67) and (2) Cell Health Profiling which enables quantification of markers expressed in the cell body and dendrites (DCX, MAP2 and CC3). Finally, the overall number of BrdU, Ki67, DCX, MAP2 and CC3 positive cells over DAPI positive cells were quantified.

### 2.7 Statistical analysis

Analysis were performed using IBM SPSS statistical software version 22 and StataCorp STATA version 13. Non-parametric Mann-Whitney U and Wilcoxon test, and Chi-squared test were used to investigate differences in the demographics of depressed and non-depressed patients, and for differences in the expression of proliferation and differentiation markers between depressed and non-depressed, at baseline and TW4. Spearman’s r_s_ test was used to investigate correlations among the markers. Binary logistic regression was used when assessing the predictive effect of TW0 and TW4 immunostaining outcomes with later IFN-α–induced depression.

## 3 Results

Supplementary data associated with this article can be found, in the online version, at https://doi.org/10.1016/j.bbi.2019.06.018.

Details on descriptive statistics (mean ± SD) and analysis of differences for each marker at TW0 and TW4, comparing depressed with non-depressed patients, are presented in the Supplementary Table 1. Notably, patients who developed IFN-α–induced depression had a significantly higher number of apoptotic (CC3+ and CC3/Brdu+) cells at TW0, but not at TW4, and a significantly lower number of neuroblast (DCX+) cells at TW4, but not at TW0 (Supplementary Table 1). While serum from all patients increased the number of DCX+ cells and neuronal (MAP2+) cells between TW0 and TW4, patients who developed IFN-α–induced depression had a significantly lower increase in the number of DCX+ cells between TW0 and TW4 (Supplementary Table 1).

**Supplementary Table 1:** 

Of note, all patients developed depression between TW8 and TW24 (Table 2); thus, our analysis focused on predicting the development of (later) IFN-α–induced depression, through a series of logistic regressions.

### 3.1 Higher baseline apoptosis predicts later IFN-α–induced depression

#### 3.1.1 Proliferation and apoptosis

**Fig. 1:**
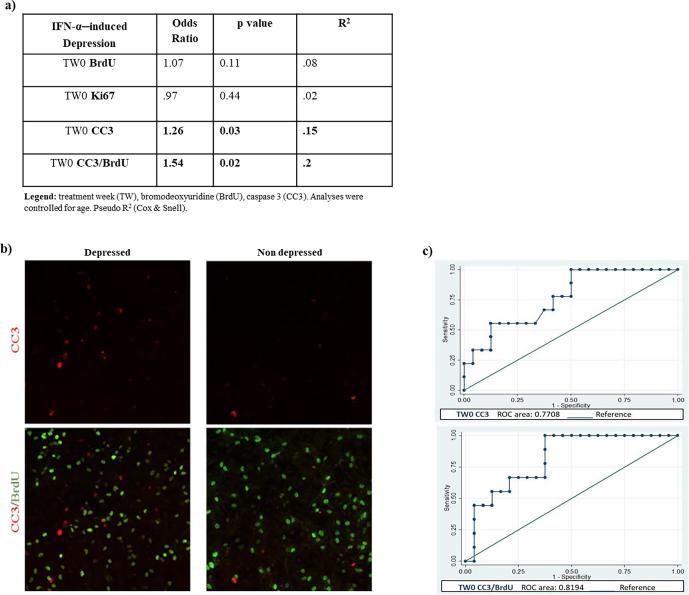
Baseline markers of proliferation and apoptosis as predictors of interferon-alpha (IFN-α)–induced depression. Main predictive outcomes of baseline proliferating and apoptotic markers on later IFN-α–induced depression (a). Representative images of cells treated with baseline serum from depressed and non-depressed patients. Apoptotic cells were stained with caspase 3 (CC3) (red labelling), and apoptotic cells expressing the proliferating marker bromodeoxyuridine (BrdU) (green labelling) were identified as CC3/BrdU (b). Receiver Operating Characteristic (ROC) curves of baseline CC3 and CC3/BrdU predictors of IFN-α–induced depression (c). (For interpretation of the references to colour in this figure legend, the reader is referred to the web version of this article.)

We first assessed whether any changes in proliferating or apoptotic markers, detected upon treatment with TW0 serum samples, were able to predict later IFN-α–induced depression. When entering separately each marker in the model, results show no effect of baseline proliferating markers (BrdU and Ki67) in predicting IFN-α–induced depression (Fig. 1a). However, a baseline higher number of apoptotic CC3+ cells, and of proliferating cells undergoing apoptosis, CC3/BrdU+ cells, significantly predicted depression. In particular, for a one-unit increase in the number of TW0 CC3+ cells, and of TW0 CC3/BrdU+ cells, there is respectively 26% and 54% increase in the odds of becoming depressed (respectively, odds ratio = 1.26, p = 0.03; odds ratio = 1.54, p = 0.02; Fig. 1a and b).

Subsequently, in order to test the accuracy of the model with CC3 and CC3/BrdU as predictors, we plotted the true positive values (Specificity) against the false positive values (Sensitivity) using the Receiver Operating Characteristic (ROC) curve. Since CC3 is highly correlated with CC3/BrdU (r_s_ = 0.9, p < 0.0001), we generated two independent models and ROC curves. Results show that the models with TW0 CC3 and TW0 CC3/BrdU as variables are respectively 77% and 81% accurate in predicting later development of depression (Fig. 1c).

#### 3.1.2 Differentiation and apoptosis

Having shown the significant role of changes in the number of CC3+ cells caused by treatment with baseline serum samples in predicting IFN-α–induced depression, we subsequently investigated whether changes in the neurogenic markers DCX and MAP2, as well as the apoptotic marker CC3 detected during the differentiation stage, were predictive of later IFN-α–induced depression. When entering separately each marker in the model, results show no significant effect for any of those markers (Table 3).

**Table 3:** Baseline neurogenic and apoptotic markers as predictors of interferon-alpha (IFN-α)–induced depression.

**IFN-α–induced Depression**	**Odds Ratio**	**p value**	**R^2^**
TW0 **DCX**	1.08	0.3	0.03
TW0 **MAP2**	1.08	0.4	0.02
TW0 **CC3**	1.03	0.6	0.006
TW0 **CC3/MAP2**	0.91	0.7	0.003

Treatment week (TW), doublecortin (DCX), microtubule associated protein 2.

(MAP2), caspase 3 (CC3). Analyses were controlled for age. Pseudo R^2^ (Cox & Snell).

### 3.2 Lower neurogenesis at TW4 predicts later IFN-α–induced depression

#### 3.2.1 Proliferation and apoptosis

As for data at baseline, we then investigated whether any changes in proliferating (BrdU and Ki67) or apoptotic (CC3) markers, detected upon treatment with TW4 serum samples, were able to predict later IFN-α–induced depression. When entering separately each marker in the model, results show no significant effect for any of those markers (Table 4).

**Table 4:** Treatment week 4 (TW4) proliferating and apoptotic markers as predictors of interferon-alpha (IFN-α)–induced depression.

**IFN-α-induced Depression**	**Odds Ratio**	**p value**	**R^2^**
TW4 **BrdU**	1.02	0.3	0.03
TW4 **Ki67**	0.93	0.1	0.08
TW4 **CC3**	1.05	0.5	0.01
TW4 **CC3/BrdU**	1.01	0.9	0.0

Treatment week (TW), bromodeoxyuridine (BrdU), caspase 3 (CC3).

Analyses were controlled for age. Pseudo R^2^ (Cox & Snell).

#### 3.2.2 Differentiation and apoptosis

**Fig. 2:**
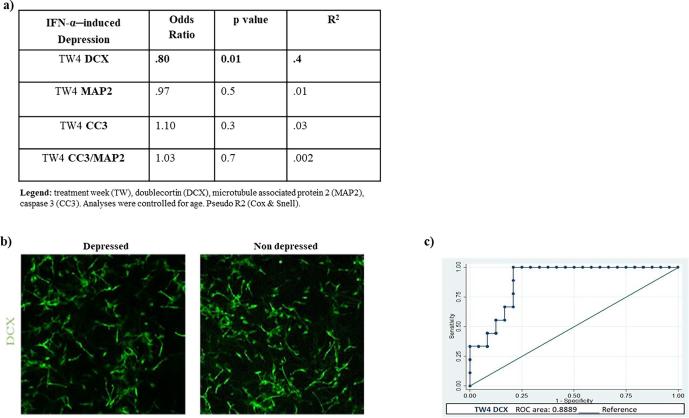
Treatment week 4 (TW4) neurogenic and apoptotic markers as predictors of IFN-α–induced depression. Main predictive outcomes of TW4 neurogenic and apoptotic markers on later IFN-α–induced depression (a). Representative images of cells treated with TW4 serum from depressed and non-depressed patients. Neuroblasts were stained with doublecortin (DCX) (green labelling) (b). Receiver Operating Characteristic (ROC) curve of TW4 DCX predictor of IFN-α–induced depression (c).(For interpretation of the references to colour in this figure legend, the reader is referred to the web version of this article.)

Secondly, we assessed whether changes in neurogenic (DCX and MAP2) and apoptotic markers (CC3) detected upon treatment with TW4 serum samples were able to predict later depression. When entering separately each marker in the model, results show that for a one-unit decrease in TW4 DCX+ cells, there is a 20% increment in the odds of becoming depressed (odds ratio = 0.80; p = 0.01; Fig. 2a and b). In addition, a ROC curve was generated showing that the model with TW4 DCX as variable is 88% accurate in predicting later development of depression (Fig. 2c).

### 3.3 A lower increase in neurogenesis between baseline and TW4 predicts IFN-α–induced depression

#### 3.3.1 Proliferation and apoptosis

Having shown that *baseline* apoptotic markers are predictors of later IFN-α–induced depression, we subsequently investigated whether also *changes* between TW0 and TW4 in the number of proliferating and apoptotic cells were predictive of later depression. As before, we focussed on two proliferating markers BrdU and Ki67, and on the apoptotic marker CC3. Results show no significant effect for any of those markers on later development of depression (Table 5).

**Table 5:** Change in proliferating and apoptotic markers between treatment week (TW0) and TW4 as predictor of interferon-alpha (IFN-α)–induced depression.

**IFN-α–induced Depression**	**Odds Ratio**	**p value**	**R^2^**
Δ_TW4-TW0_**BrdU**	0.91	0.82	0.002
Δ_TW4-TW0_**Ki67**	0.93	0.61	0.01
Δ_TW4-TW0_**CC3**	0.95	0.36	0.03
Δ_TW4-TW0_**CC3/BrdU**	0.7	0.055	0.13

Treatment week (TW), bromodeoxyuridine (BrdU), caspase 3 (CC3).

Analyses were controlled for age. Pseudo R^2^ (Cox & Snell).

#### 3.3.2 Differentiation and apoptosis

As before, we then investigated whether *changes* between TW0 and TW4 also in the number of neurogenic and apoptotic markers were predictive of later depression.

As mentioned above, DCX+ cells overall tended to increase when cells were treated with TW4 serum compared with TW0 serum; however, patients who developed IFN-α–induced depression had a significantly lower increase in the number of DCX+ cells between TW0 and TW4 (see Supplementary Table 1).

**Fig. 3:**
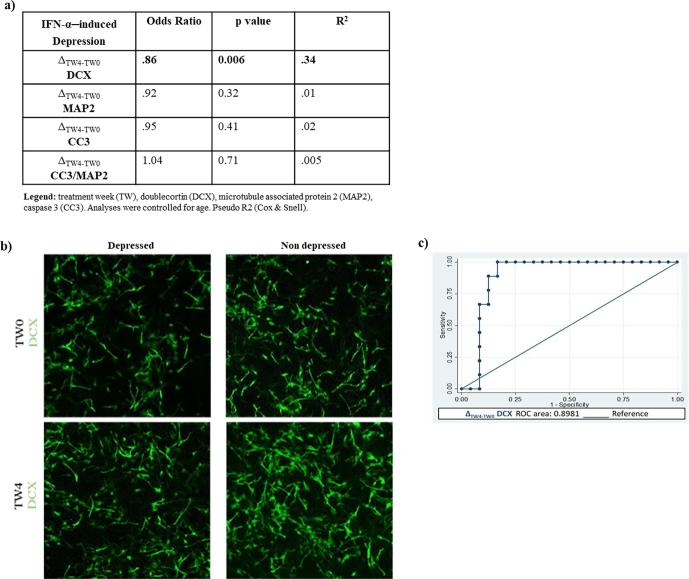
Changes in neurogenic and apoptotic markers between treatment week 0 (TW0) and TW4 as predictors of IFN-α–induced depression. Main predictive outcomes of differences in neurogenic and apoptotic markers between TW0 and TW4 on later IFN-α–induced depression (a). Representative images of cells treated with TW0 and TW4 serum from depressed and non-depressed patients. Neuroblasts were stained with doublecortin (DCX) (green labelling) (b). Receiver Operating Characteristic (ROC) curve of Δ_TW4-TW0_ DCX predictor of IFN-α–induced depression (c).

When entering separately each marker in the model, results show that a lower increase in DCX+ cells between TW0 and TW4 is predictive of later development of depression. In particular, for a one-unit lower in the number of Δ_TW4-TW0_ DCX+ cells, there is a 14% increment in the odds of becoming depressed (odds ratio = 0.86, p = 0.006; Fig. 3a and b). In addition, a ROC curve was generated showing that the model with Δ_TW4-TW0_ DCX as variable is 89% accurate in predicting later development of depression (Fig. 3c).

### 3.4 Low neurogenesis at TW4 and a lower increase in neurogenesis between baseline and TW4 most accurately predict IFN-α–induced depression

**Fig. 4:**
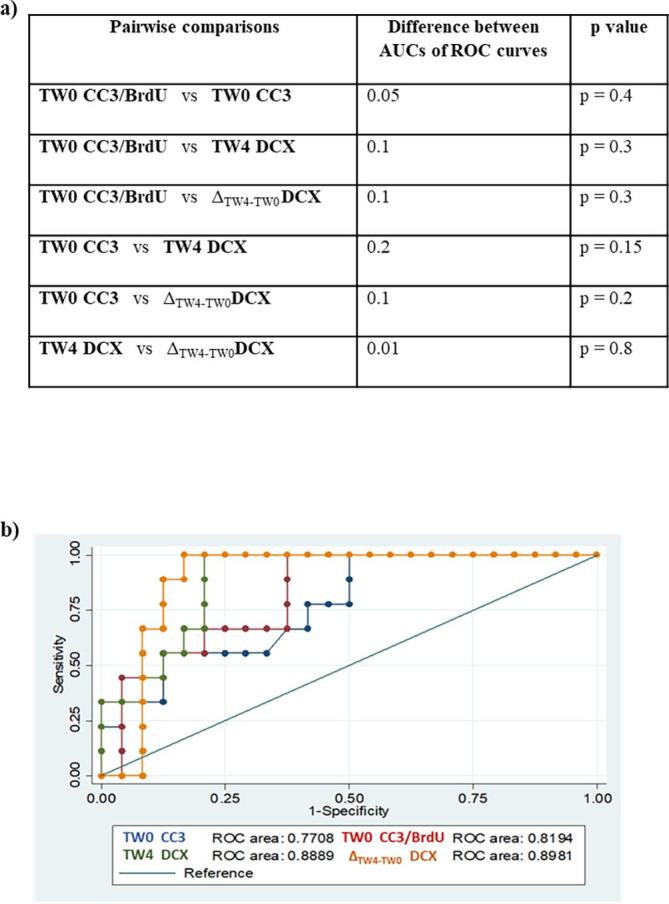
Best accurate model of prediction of interferon-alpha (IFN-α)–induced depression. Pairwise comparisons of Areas Under the Curves (AUCs) of Receiver Operating Characteristic (ROC) curves generated from treatment week 0 (TW0) and TW4 best predictive markers of IFN-α–induced depression (a). Summary graph containing ROC curves of TW0 and TW4 best predictors of IFN-α–induced depression (b).

Finally, having discovered that a high number of TW0 CC3+, TW0 CC3/BrdU+, and a low number of TW4 DCX+ and Δ_TW4-TW0_ DCX+ cells, were significantly predictive of IFN-α–induced depression, we subsequently investigated which model (containing those markers as variables) could be defined as the best one in terms of accuracy when predicting IFN-α–induced depression. Pairwise analysis comparing ROC curves previously generated from the apoptotic (TW0 CC3 and TW0 CC3/BrdU) and neurogenic (TW4 DCX and Δ_TW4-TW0_ DCX) markers do not show any significant difference in the accuracy of predicting IFN-α–induced depression (respectively, 77%, 81%, 88% and 89% accuracy; Fig. 4a and b), even if numerically the TW4 DCX and the Δ_TW4-TW0_ DCX have the highest predictive values.

## 4 Discussion

Using a model of inflammation-induced depression, in this study we provide the first evidence that blood factors, contained in the systemic milieu of patients receiving chronic treatment with IFN-α, can alter cell apoptosis and the neurogenic process *in vitro,* and potentially activate cellular processes that ultimately may contribute to the development of IFN-α–induced depression. In particular, we demonstrated that higher apoptosis upon treatment with baseline (TW0) serum, lower neurogenesis upon treatment with TW4 serum, and a lower increase in neurogenesis between treatments with TW0 and TW4 serum, are predictive of later development of depression – the last two models with, respectively, 88% and 89% accuracy.

Firstly, our findings indicate that baseline peripheral factors can be predictors of depression when measuring their impact on HPC apoptosis. Indeed, although none of the proliferating and neurogenic markers were affected upon treatment with baseline samples, a high number of cells positive for the apoptotic marker CC3 was predictive of later depression. However, using a larger cohort of IFN-α treated HCV patients (from which we have selected all serum samples used in the present study) we have found no differences at TW0 in the levels of key inflammatory cytokines, including IL-1β, IL-6, IL-17 and TNF-α, between those who later develop or do not develop depression (although, interestingly, we found differences in the prediction of fatigue) (Hepgul et al., 2016, Russell et al., 2019). This lack of difference in ‘usual suspect’ cytokines suggests the involvement of other factors contained in serum from depressed patients that are able to contribute to the increase in cell apoptosis detected in our *in vitro* study. Indeed, our transcriptome analysis conducted in the same larger cohort of IFN-α treated HCV patients revealed the presence of a distinct set of genes differentially expressed at TW0 between depressed and non-depressed patients (Hepgul et al., 2016). Among them, ubiquitin-fold modifier 1 (UFM1) and eukaryotic translation initiation factor 4B (EIF4B) were highly upregulated in patients who developed IFN-α–induced depression (Hepgul et al., 2016). UFM1 and EIF4B gene belong to respectively, the oxidative stress response signalling and the rapamycin signalling pathway, which are associated with regulation of apoptosis, and synaptic plasticity (Chen et al., 2012, Narasimhan et al., 2012), therefore potentially involved in the detrimental effect seen on cell death upon treatment with serum from depressed patients.

In this study we also demonstrate that lower levels of neurogenesis upon treatment with serum at TW4, and a lower increase in neurogenesis between TW0 and TW4, are predictive of later development of depression. Similarly to baseline, results from cytokines analysis conducted in samples from our larger cohort of IFN-α treated HCV patients, and collected at TW4, did not reveal any differences in the expression of the candidate cytokines previously mentioned, either as absolute values at TW4 or as increased between TW0 and TW4 when comparing depressed with non-depressed (Hepgul et al., 2016, Russell et al., 2019). However, data from our aforementioned transcriptome analysis show that, at TW4, patients who developed depression had a higher expression of a distinct set of genes, including cyclin dependent kinase 5 (CDK5) and CDK8, as well as insulin-like growth factor-binding protein 5 (IGFBP5) and IGFBP7 (Hepgul et al., 2016), which are known to regulate both cell proliferation and differentiation (Alexander et al., 2004, Tanno et al., 2005). Furthermore, pathway analysis conducted in our larger cohort of IFN-α treated patients showed that distinct neuroplasticity-related pathways, including extracellular signal-regulated kinase 5 (ERK5) signaling were modulated in blood of depressed patients at TW4, when compared with baseline (Hepgul et al., 2016). ERK5 is a member of the mitogen-activated protein kinase (MAPK) family, that includes ERK1/2, p38, and JNK, and is known to regulate neurogenesis (Wang et al., 2014) and to participate in the neuronal modulation of depression (Todorovic et al., 2009).

We were surprised that treatment with serum collected after 4 weeks of IFN-α treatment (TW4) was able to increase neurogenesis, since *in vitro* we found that IFN-α can detrimentally affect neuronal formation (Borsini et al., 2017c). On possible explanation could be due to the activation of distinct compensatory mechanisms, which might have prevented significant damage caused by exposure to serum from both depressed and non-depressed. Further characterisation of the cellular mechanisms underlying the effect of IFN-α on neurogenesis are required, especially upon a longer exposure to IFN-α, in order to identify potential neuroprotective mechanisms activated only at a later stage during the treatment.

Of course, this immortalized cell line we used in the study, while being of invaluable importance for our understanding of molecular mechanisms occurring in the hippocampal progenitor cells, may differ from the scenario of an adult *in vivo* environment and the adult neurogenic niche, in particular because of the absence of microglia cells, which are well-known regulators of inflammatory signaling pathways. However, our previous studies with this *in vitro* model have been successfully replicated in animal studies, including changes in neurogenesis by cortisol, IL-1 and antidepressants, and changes in stress- and antidepressants-regulated genes (Anacker et al., 2013a, Anacker et al., 2013b, Anacker et al., 2011, Borsini et al., 2017b, Horowitz et al., 2014, Zunszain et al., 2012). Therefore, we are confident that our results are relevant to the human brain.

The number of samples is also very small and collected primarily from male patients, of whom we have relatively few information with respect to their clinical profile. Therefore, the predictive models presented here should be replicated in a large cohort of patients. However, this was an extremely difficult group of patients to recruit and furthermore, the vast majority of patients recruited had to be excluded due to our strict criteria (see Methods). These strict criteria however allowed us to eliminate potential confounding factors, which otherwise might have affected the results. Finally, no control or placebo group was used for this study. However, the main aim in this case was not to test the effect of IFN-α on later depression, but instead to identify novel biological predictors for the development of depression. As such, we used serum samples of patients who did develop IFN-α-induced depression in comparison with serum samples of patients who did not develop IFN-α-induced depression.

## 5 Conclusions

In summary, our *in vitro* study demonstrates that hippocampal progenitor cells can be regulated by treatment with serum from IFN-α–treated HCV patients, and that subsequent alteration in neurogenesis and in cell survival is predictive of later development of depression. We believe that these findings may shed light on the pathogenesis of the depressive disorder, and further propose that the set-up employed in the present *in vitro* study, using a human HPCs experimental model together with a medium-throughput serum screening assay, offers an excellent opportunity for identification of novel therapeutic strategies for treatment of neuropsychiatric disorders.
