# Supplementary material for: The role of circulatory systemic environment in predicting interferon-alpha–induced depression: The neurogenic process as a potential mechanism
Source: Brain Behav Immun. 2019 Oct;81:220–7. doi: 10.1016/j.bbi.2019.06.018 (PMC6934231; doi:10.1016/j.bbi.2019.06.018)
Supplement: Supplementary file 1 [file mmc1.pdf]

Supplementary Materials

Table 1. Differences in the percentage of proliferation and differentiation markers over DAPI positive cells, between depressed and non-depressed patients at baseline and treatment week 4.

**Markers**	**TW0** **Depressed** (mean % ± SD)	**TW0** **Non-Depressed** (mean % ± SD)	**TW0** **Depressed** vs **Non-Depressed** (p value)	**TW4** **Depressed** (mean % ± SD)	**TW4** **Non-Depressed** (mean % ± SD)	**TW4** **Depressed** vs **Non-Depressed** (p value)	**ΔTW4-TW0** **All patients** (p value)	**ΔTW4-TW0** **Depressed** vs **Non-Depressed** (p value)
**Proliferation**								
BrdU	33.9 ± 10.6	27.7 ± 8.8	p=0.1*	36.7 ± 13.3	31.5 ± 13.2	p=0.5*	p=0.2**	p=0.7*
Ki67	74.3 ± 13.0	77.8 ± 11.3	p=0.5*	71.1 ± 11.9	77.7 ± 8.9	p=0.1*	p=0.8**	p=0.6*
CC3	9.1 ± 3.9	5.4 ± 3.7	**p=0.02***	9.6 ± 6.2	8.3 ± 4.5	p=0.8*	p=0.1**	p=0.5*
CC3/BrdU	5.3 ± 2.3	2.8 ± 2.1	**p=0.004***	3.6 ± 2.1	3.6 ± 2.4	p=0.8*	p=0.9**	p=0.052*
**Differentiation**								
DCX	14.9 ± 5.3	12.7 ± 5.0	p=0.3*	22.0 ± 5.0	37.5 ± 10.7	**p<0.0001***	**p<0.0001****	**p=0.001***
MAP2	14.1 ± 3.7	12.5 ± 5.0	p=0.5*	26.7 ± 12.6	29.1 ± 9.9	p=0.7*	**p<0.0001****	p=0.3*
CC3	6.7 ± 4.7	6.0 ± 4.3	p=0.6*	8.7 ± 3.1	7.0 ± 4.4	p=0.1*	p=0.1**	p=0.2*
CC3/MAP2	2.2 ±1.0	2.5 ± 2.2	p=0.6*	4.2 ± 2.0	3.9 ± 3.3	p=0.2*	**p=0.02****	p=0.8*

Legend: treatment week (TW), bromodeoxyuridine (BrdU), caspase 3 (CC3), doublecortin (DCX), microtubule associated protein 2 (MAP2). *Mann-Whitney U test, ** Wilcoxon test.
